# Effect of two non-synonymous ecto-5′-nucleotidase variants on the genetic architecture of inosine 5′-monophosphate (IMP) and its degradation products in Japanese Black beef

**DOI:** 10.1186/s12864-017-4275-4

**Published:** 2017-11-13

**Authors:** Yoshinobu Uemoto, Tsuyoshi Ohtake, Nanae Sasago, Masayuki Takeda, Tsuyoshi Abe, Hironori Sakuma, Takatoshi Kojima, Shinji Sasaki

**Affiliations:** 1 National Livestock Breeding Center, Nishigo, Fukushima, 961-8511 Japan; 2 Present address: Graduate School of Agricultural Science, Tohoku University, Sendai, Miyagi 980-0845 Japan

**Keywords:** GWAS, IMP, Japanese Black cattle, Meat quality, NT5E

## Abstract

**Background:**

*Umami* is a Japanese term for the fifth basic taste and is an important sensory property of beef palatability. Inosine 5′-monophosphate (IMP) contributes to *umami* taste in beef. Thus, the overall change in concentration of IMP and its degradation products can potentially affect the beef palatability. In this study, we investigated the genetic architecture of IMP and its degradation products in Japanese Black beef. First, we performed genome-wide association study (GWAS), candidate gene analysis, and functional analysis to detect the causal variants that affect IMP, inosine, and hypoxanthine. Second, we evaluated the allele frequencies in the different breeds, the contribution of genetic variance, and the effect on other economical traits using the detected variants.

**Results:**

A total of 574 Japanese Black cattle were genotyped using the Illumina BovineSNP50 BeadChip and were then used for GWAS. The results of GWAS showed that the genome-wide significant single nucleotide polymorphisms (SNPs) on BTA9 were detected for IMP, inosine, and hypoxanthine. The *ecto-5′-nucleotidase (NT5E)* gene, which encodes the enzyme NT5E for the extracellular degradation of IMP to inosine, was located near the significant region on BTA9. The results of candidate gene analysis and functional analysis showed that two non-synonymous SNPs (c.1318C > T and c.1475 T > A) in *NT5E* affected the amount of IMP and its degradation products in beef by regulating the enzymatic activity of NT5E. The *Q* haplotype showed a positive effect on IMP and a negative effect on the enzymatic activity of NT5E in IMP degradation. The two SNPs were under perfect linkage disequilibrium in five different breeds, and different haplotype frequencies were seen among breeds. The two SNPs contribute to about half of the total genetic variance in IMP, and the results of genetic relationship between IMP and its degradation products showed that *NT5E* affected the overall concentration balance of IMP and its degradation products. In addition, the SNPs in *NT5E* did not have an unfavorable effect on the other economical traits.

**Conclusion:**

Based on all the above findings taken together, two non-synonymous SNPs in *NT5E* would be useful for improving IMP and its degradation products by marker-assisted selection in Japanese Black cattle.

**Electronic supplementary material:**

The online version of this article (doi: 10.1186/s12864-017-4275-4) contains supplementary material, which is available to authorized users.

## Background

Beef palatability is one of the most economically important objectives for the breeding of Japanese Black cattle, whose beef has a unique characteristic of intense marbling. The palatability of beef is primarily evaluated based on sensory characteristics such as taste, tenderness, juiciness, aroma, and so on. However, sensory characteristics are difficult to measure, are largely subjective, and possess low heritability [1, 2]. Therefore, it is difficult to genetically improve the quality of meat by relying on sensory characteristics alone. Therefore, another approach to incorporate indicators of these sensory characteristics is necessary.

*Umami* is a Japanese term for the fifth basic taste and is an important sensory property of foods, along with many other characteristics, including texture and flavor [3]. Inosine 5′-monophosphate (IMP) is a major nucleotide in postmortem muscle and contributes to the taste and flavor in meat [4]. The combination of IMP and glutamic acid or aspartic acid enhances the *umami* taste, and is known as *umami* intensity [5]. Recently, Suzuki et al. [6] reported that the ‘strength aroma’ and ‘*umami* intensity’ based on the panel test contributed to the overall taste evaluation in seven beef brands of Japanese Black beef, and the amount of IMP was significantly correlated with the ‘*umami* intensity’ of the panel test. The degradation products of IMP (inosine and hypoxanthine) are also important indicators of beef palatability. Inosine and hypoxanthine do not contribute to *umami* taste in beef, but hypoxanthine, in combination with some amino acids and peptides, may contribute to bitterness in meat [7]. Therefore, the overall change in concentration of IMP and its degradation products can potentially affect the beef palatability. Therefore, a genetic understanding of IMP and its degradation products is important in beef cattle breeding to enhance the meat quality. The heritability estimates of glutamic acid and aspartic acid in beef were low (0.17 and 0.00, respectively) but those of IMP, inosine, and hypoxanthine were low to moderate (0.48, 0.33, and 0.23, respectively) in Japanese Black cattle population [8].

**Fig. 1:**
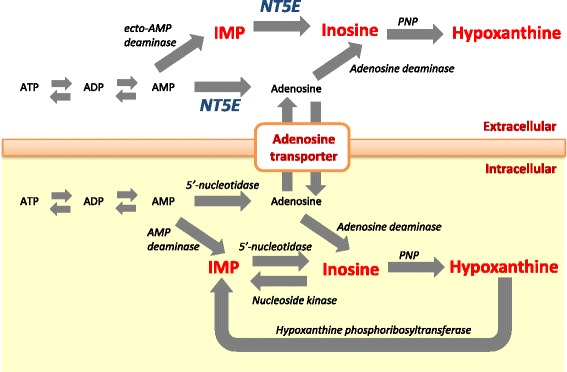
Schematic representation of the pathway of formation and degradation of inosine 5′-monophosphate (IMP) in muscle. Each abbreviation is adenosine triphosphate (ATP), adenosine diphosphate (ADP), adenosine 5′-monophosphate (AMP), ecto-5′-nucleotidase (NT5E), and purine nucleoside phosphorylase (PNP). Black and red characters indicate products, and blue and black characters in italics indicate enzymes. This schematic representation is based on previous reports [9–12]

During the pathway of the formation and degradation of IMP in muscles, Adenosine triphosphate (ATP) is rapidly degraded to adenosine diphosphate (ADP) and adenosine monophosphate (AMP), which is then degraded to IMP. The IMP is further hydrolyzed to inosine by the enzymes of 5′-nucleotidase in intracellular and ecto-5′-nucleotidase (NT5E) in extracellular, which, in turn, is degraded to hypoxanthine (Fig. 1) [9–12]. The aging period after slaughter induces structural changes in the cell membrane, leading to intracellular water efflux [13]. Some of the enzymes involved in the degradation of IMP are gradually inactivated during the aging period of beef [14, 15]. Therefore, there is a strong biochemical relationship between IMP and its degradation products in beef, and detailed information on the genetic architecture of these traits is necessary to improve meat quality by using these traits as indicators. However, detailed studies on the genetic architecture of these traits and their genetic relationship have, to our knowledge, not been performed.

To investigate the genetic architecture of IMP and its degradation products in Japanese Black beef, we performed genome-wide association studies (GWAS) and functional analysis to detect the causal variants that affect IMP and its degradation products at first. Second, we evaluated the allele frequencies in the different breed populations, the contribution of genetic variance, and the effect on other economical traits using the detected variants. This study identified quantitative trait nucleotides (QTNs) in *ecto-5′-nucleotidase (NT5E)*, which serves as a key enzyme for IMP and it degradation products.

## Results

### GWAS

**Fig. 2:**
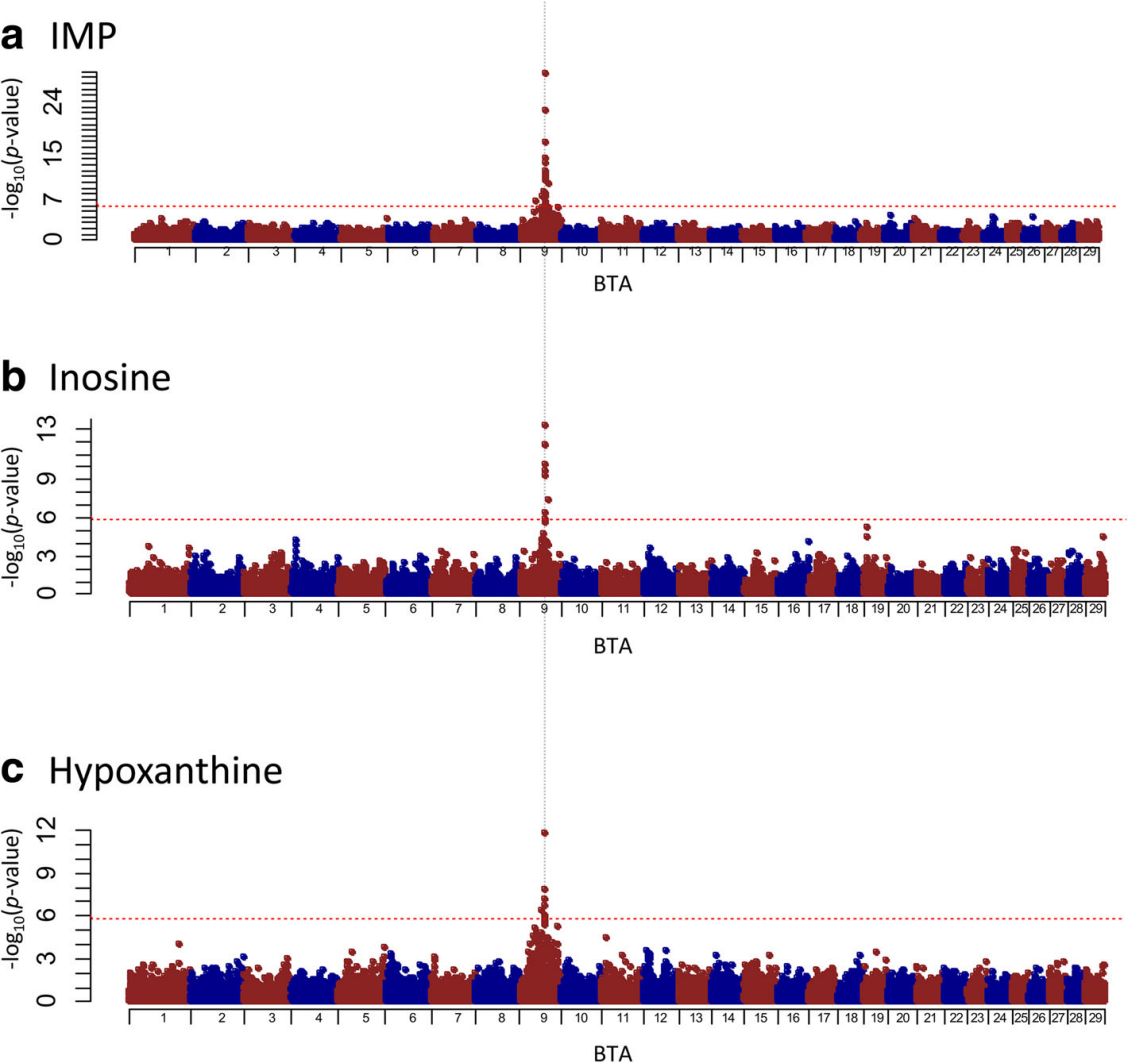
Genome-wide plots of *p*-values (˗log_10_) for significantly associated loci. **a** Inosine 5′-monophosphate (IMP), **b** Inosine, and **c** Hypoxanthine. The *x*-axis indicates the chromosome number, and the *y*-axis indicates *p*-values (˗log_10_). Dashed red line indicates the threshold of the Bonferroni 5% significance level

**Fig. 3:**
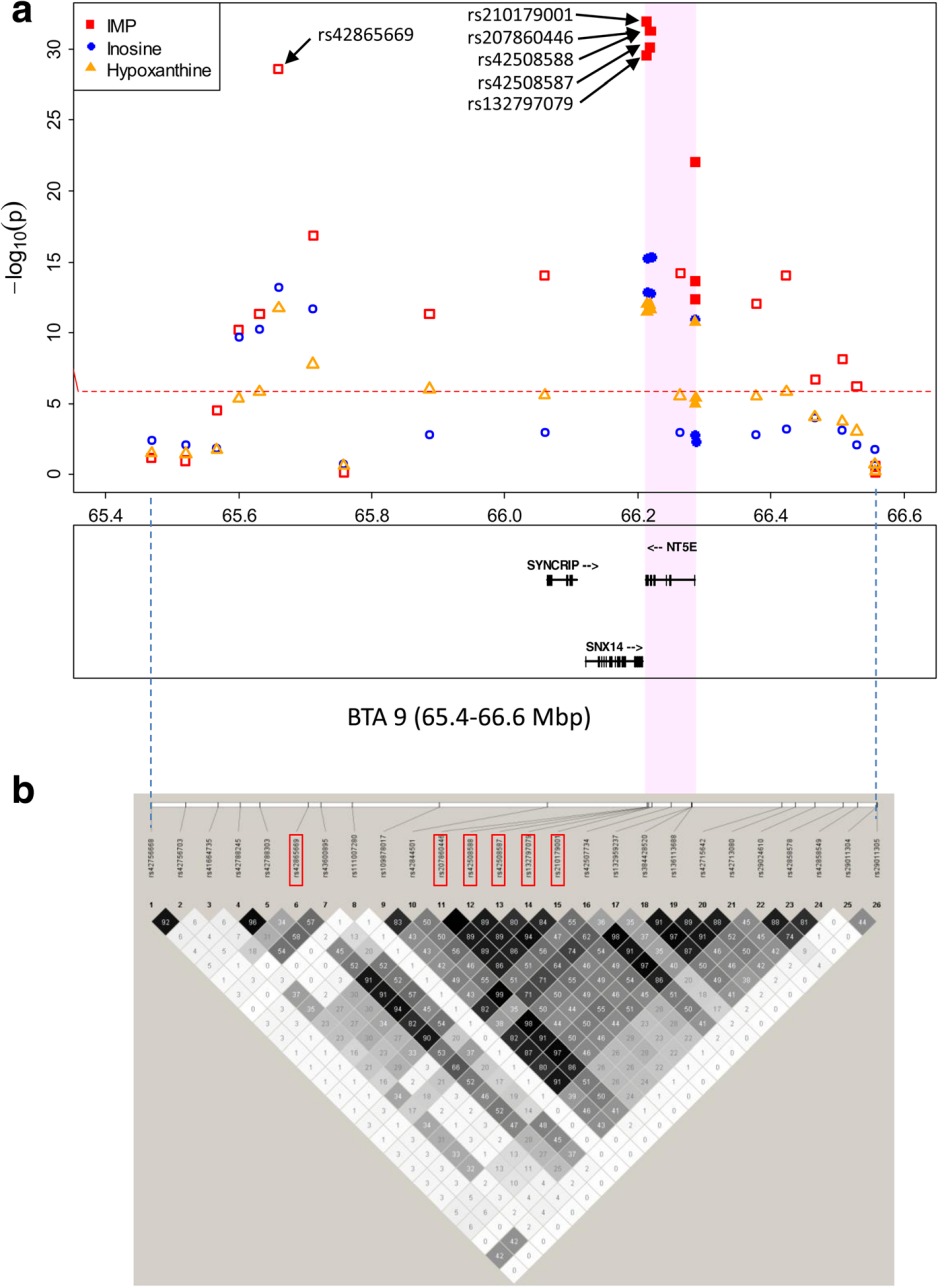
The significant region and linkage disequilibrium (LD) from 65.4 to 66.6 Mbp on BTA 9. **a** The regional plots of the locus are associated with inosine 5′-monophosphate (IMP), inosine, and hypoxanthine. The *x*-axis indicates the Mbp, and the *y*-axis indicates *p*-values (˗ log_10_). The gene loci and their strand were annotated based on Btau4.6 assembly from the bovine genome database (http://bovinegenome.org/). The dashed red line indicates the threshold of the Bonferroni 5% significance level. The *p*-values of SNPs on the SNP array (unfilled points) and detected variants in *ecto-5′-nucleotidase (NT5E)* (filled points) were plotted. **b** LD coefficients (r^2^) between the SNPs in this region. Black fields display r^2^ values >0.80, and white and gray fields display r^2^ values <0.80

A total of 574 Japanese Black cattle with records reported by Sakuma et al. [8] and BovineSNP50 genotypes reported by Sasago et al. [16] were used. The 5% genome-wide significant threshold was accounted for in multiple testing via Bonferroni correction with *p*-value = 1.37 × 10^˗6^. The results of GWAS are shown in Fig. 2 and Additional file 1: Table S1. The significant SNPs associated with the three phenotypes were detected on BTA 9. Specifically, the rs42865669 SNP on BTA 9 had the highest significance in IMP (*p*-value = 2.8 × 10^−29^), inosine (*p*-value = 6.7 × 10^−14^), and hypoxanthine (*p*-value = 1.8 × 10^−12^). The rs42865669 SNP was not located within any genes, but was located about 500 kb from *NT5E* (Fig. 3a). *NT5E* encodes a membrane-bound enzyme for extracellular degradation of AMP to adenosine and degradation of IMP to inosine (Fig. 1). Thus, the *NT5E* could be regarded as the positional candidate gene for IMP and its degradation products. Based on the results from our GWAS, we performed candidate gene analysis on detecting the variants in *NT5E* and on testing the association between the variants in *NT5E* and these traits.

### NT5E sequencing and association test

**Fig. 4:**
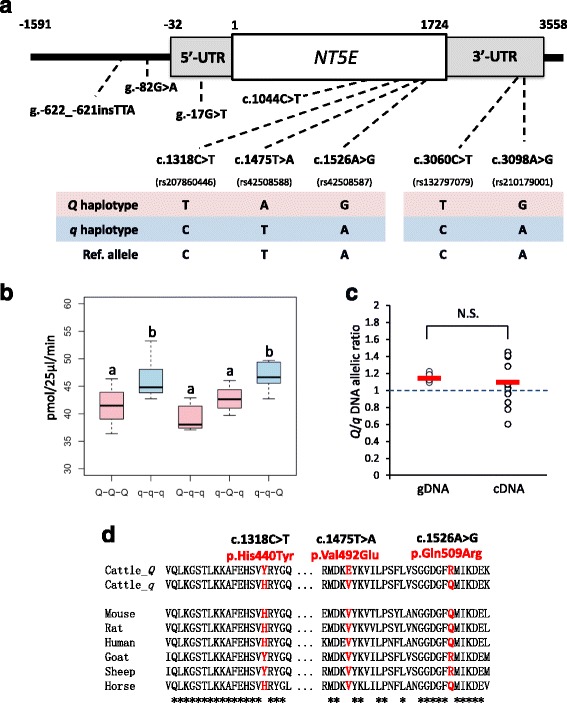
The schematic structure and the SNP features of *ecto-5′-nucleotidase* (*NT5E*). **a** Schematic representation of the positions of variants from the 5′-upstream region to the 3′-UTR in *NT5E*. The detailed positions and names of the variants are shown in Table 1. The *Q* and *q* haplotypes are defined by the genotypes of the three non-synonymous SNPs on exon 7 and exon 8 and two SNPs on 3′-UTR of exon 9. The *Q* haplotype has a positive effect on inosine 5′-monophosphate (IMP). Bovine reference (Ref) allele from Gene Bank accession no. NM_174129.3 is also shown. **b** The IMPase activity of ecto-5′-nucleotidase in COS-7 cells. Q-Q-Q, the construct with *Q* haplotype; q-q-q, the construct with *q* haplotype; Q-q-q, the construct mutated from *q* haplotype to *Q* allele in c.1318C > T; q-Q-q, the construct mutated from *q* haplotype to *Q* allele in c.1475 T > A; q-q-Q, the construct mutated from *q* haplotype to *Q* allele in c.1526A > G. The superscript letters indicate significant differences among five constructs tested by analysis of variance followed by a Tukey HSD (honestly significant difference) multiple comparison test (*p*-value <0.05). **c** The allelic imbalance test for levels of *NT5E* mRNA in the heterozygotes. The *y*-axis shows the ratio of peak height of the *Q* allele over the *q* allele in the genomic DNA (gDNA) and the complementary DNA (cDNA) from the same animal. Red bars indicate the mean expression. No significant (N.S.) difference was shown between them. **d** Multiple sequence alignment of the regions flanking p.His440Tyr, p.Val492Glu, and p.Gln509Arg. *Q* haplotype and *q* haplotype sequences of the cattle are shown on the top and other mammalian sequences are shown below

To detect variants in *NT5E*, we determined the nucleotide sequences of all of the exons and the proximal promoter region by direct sequencing. The variants detected are shown in Table 1. Eight SNPs and an insertions and deletions variant (indel) were detected. These variants were present in the dbSNP database. Three non-synonymous SNPs, an indel, two SNPs in 5′-upstream/5′-untransrated region (UTR), and two SNPs in 3′-UTR were genotyped in all animals. The association tests of these variants with three traits were performed, and the results are shown in Table 1 and Fig. 3a. The results showed that the three non-synonymous SNPs (c.1318C > T in the exon 7, c.1475 T > A, and c.1526A > G in the exon 8) and the two SNPs in 3′-UTR of exon 9 (c.3060C > T and c.3098A > G) had high significance in IMP (*p*-value = 3.0 × 10^−30^ to 1.1 × 10^−32^), inosine (*p*-value = 1.5 × 10^−13^ to 4.6 × 10^−16^), and hypoxanthine (*p*-value = 3.2 × 10^−12^ to 9.6 × 10^−13^). These SNPs were under high linkage disequilibrium (LD) in this population, and the LD coefficients (r^2^) for the five SNPs ranged from 0.80 to 1.00 (Fig. 3b). The c.1318C > T and c.1475 T > A were under perfect LD in this population. The r^2^ values of the five SNPs and the most significant SNP (rs42865669) in the SNP array were very high (r^2^ = 0.82 to 0.91) (Fig. 3b). Among the haplotypes of five SNPs, we defined the haplotype with positive effect on IMP as *Q* haplotype and those with negative effect on IMP as *q* haplotype (Fig. 4a). The *q* haplotype had the same alleles of the *NT5E* mRNA reference sequence (NM_174129.3).

**Table 1:** The variants information in *ecto-5′-nucleotidase (NT5E)* and its association test with inosine 5′-monophosphate (IMP), inosine, and hypoxanthine in meats

Locus name^a^	refSNP variation ID	Position (bp)		Allele^a,b^		Locus	Amino Acid^c^	RAF^d,e^	*p* –value^e^		
		UMD3.1	Btau4.6	Ref	Alt				IMP	Inosine	Hypoxanthine
g.-622_-621insTTA	rs136113688	64,930,537	66,286,784	TTA	–	5′-upstream		0.44	4.2 × 10^−13^	4.1 × 10^−3^	3.9 × 10^−6^
g.-82G > A	rs384428520	64,929,995	66,286,242	G	A	5′-upstream		0.42	2.2 × 10^−14^	1.5 × 10^−3^	9.4 × 10^−6^
g.-17G > T	rs132959237	64,929,930	66,286,177	G	T	Exon 1 (5′-UTR)		0.65	8.8 × 10^−23^	9.7 × 10^−12^	1.7 × 10^−11^
c.1044C > T	rs135498711	64,860,563	66,224,147	C	T	Exon 5	p.Gly348Gly	–	–	–	–
c.1318C > T	rs207860446	64,864,545	66,220,165	C	T	Exon 7	p.His440Tyr	0.58	5.4 × 10^−32^	4.6 × 10^−16^	2.3 × 10^−12^
c.1475 T > A	rs42508588	64,866,290	66,218,420	T	A	Exon 8	p.Val492Glu	0.58	5.4 × 10^−32^	4.6 × 10^−16^	2.3 × 10^−12^
c.1526A > G	rs42508587	64,866,341	66,218,369	A	G	Exon 8	p.Gln509Arg	0.56	8.2 × 10^−31^	1.5 × 10^−13^	1.0 × 10^−12^
c.3060C > T	rs132797079	64,871,310	66,213,400	C	T	Exon 9 (3′-UTR)		0.60	3.0 × 10^−30^	5.4 × 10^−16^	3.2 × 10^−12^
c.3098A > G	rs210179001	64,871,348	66,213,362	A	G	Exon 9 (3′-UTR)		0.56	1.1 × 10^−32^	1.2 × 10^−13^	9.6 × 10^−13^

^a^Variants information in exon are based on mRNA reference sequence (GeneBank accession no. NM_174129.3). Variants information in 5′-upstream regions are based on DNA reference sequence of Btau4.6 assembly (GeneBank accession no. NC_007307)

^b^The Reference (Ref) and Altenative (Alt) alleles

^c^The protein information is based on GeneBank accession no. NP_776554.2

^d^RAF: Reference allele frequency

^e^The c.1044C > T SNP was not genotyped in all animals because of its synonymous SNP

### Functional effect of five SNPs

NT5E is the enzyme that causes the degradation of IMP and AMP on the plasma membrane to inosine and adenosine, respectively (Fig. 1). The three non-synonymous SNPs in *NT5E* could affect the protein structure and thus their enzymatic activity. To determine whether the enzymatic activity of NT5E in each haplotype is different, we transfected *NT5E*-expression plasmid into COS-7 cells. The ability to degrade IMP was examined using malachite green for the detection of the released inorganic phosphate (Pi). The results showed that the enzymatic activity of NT5E was significantly higher (*p*-value = 3.6 × 10^−4^) in constructs with *q* haplotype (q-q-q) compared to those with *Q* haplotype (Q-Q-Q) (Fig. 4b). Next, the three non-synonymous SNPs in exon 7 and 8 were mutated from *q* haplotype to *Q* allele in each locus to determine their effect on the enzymatic activity. The results showed that the activity in the construct with c.1318C > T (Q-q-q) and c.1475 T > A (q-Q-q) were detected equally well as that of Q-Q-Q (*p*-values were 0.30 and 0.88, respectively). In contrast, the activity in the construct with c.1526A > G (q-q-Q) was similar to that of q-q-q (*p*-value = 0.99), but was significantly different from those of Q-Q-Q (*p*-value = 3.7 × 10^−3^), Q-q-q (*p*-value = 8.7 × 10^−5^), and q-Q-q (*p*-value = 4.3 × 10^−2^). These results showed that c.1318C > T and c.1475 T > A in *NT5*E are the QTNs for the degradation of IMP as they affect its enzymatic activity.

The two SNPs in 3′-UTR of *NT5E* (c.1318C > T and c.1475 T > A) could potentially affect the stability of the *NT5E* transcripts. Thus, we compared the relative abundances of *Q*- versus *q*-derived *NT5E* transcripts in skeletal muscles (*n* = 14) of heterozygotes (Fig. 4c). We isolated samples of genomic DNA and complementary DNA (cDNA) from heterozygotes and then compared their allelic ratios using PeakPicker2 software [17]. The results showed that *Q*-derived *NT5E* cDNA and genomic DNA were not significantly detected as *q*-derived cDNA and genomic DNA, respectively (*p*-value = 0.51). Therefore, the two SNPs in 3′-UTR of *NT5E* did not affect the allelic imbalances of *NT5E* mRNA expression, and thus did not affect the stability of the *NT5E* transcripts.

### Bioinformatics analysis to investigate effect of non-synonymous SNPs

The multiple sequence alignments of the three amino acids based on the non-synonymous SNPs are shown in Fig. 4d. Val492 by *q* haplotype is highly conserved in mammals, and His440 and Gln509 by *q* haplotype are not conserved in some ruminants (goat and sheep), but the regions flanking His440 are highly conserved in mammals.

**Fig. 5:**
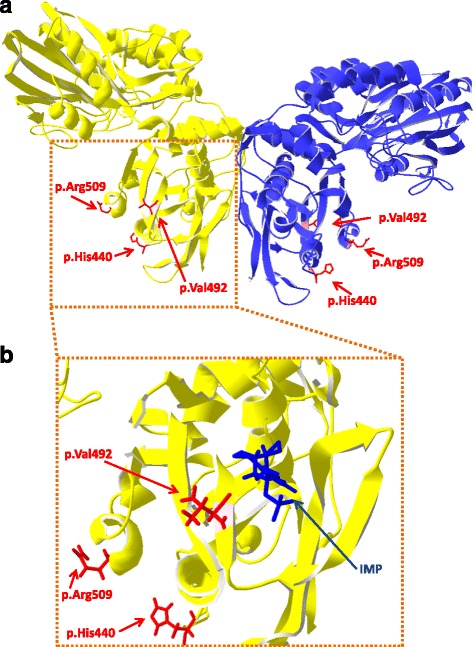
Predicted structure of ecto-5′-nucleotidase (NT5E). **a** Predicted structure of NT5E modeled by the SWISS-MODEL server [18, 19]. **b** Docking of the predicted NT5E structure with inosine 5′-monophosphate (IMP) ligand. Three amino acids based on the three non-synonymous SNPs (p.His440, p.Val492, and p.Arg509) are shown as red sticks, and IMP ligand is shown as a blue stick

To locate the three amino acids based on the non-synonymous SNPs at the protein structure of NT5E, the 3D protein structure of NT5E predicted by SWISS-MODEL server [18, 19] is illustrated in Fig. 5a. The crystal structure of human NT5E, which is known as CD73, is described a non-covalent homodimer [20], and the predicted structure of bovine NT5E using template human NT5E (4h2g) was illustrated. The docking of the predicted bovine NT5E with the IMP ligand was carried out using SWISSDOCK server [21]. One of the top five clusters for IMP ligand was located on the β-sheet where p.Val492Glu was located (Fig. 5b). In contrast, p.His440Tyr and p.Gln509Arg were not located near the binding site of the IMP ligands.

### Comparison of Q and q haplotype frequencies among five different breeds

In the functional analysis, both the SNPs (c.1318C > T and c.1475 T > A) affect the enzymatic activity and were under perfect LD in this population. To determine whether the two SNPs segregate in other breeds, they were genotyped in another 1079 animals, which were from Japanese Black cattle, Japanese Shorthorn cattle, Japanese Brown cattle, Angus cattle, and Holstein cattle. The two SNPs were under perfect LD in all the breeds. The genotype (*QQ*, *Qq*, and *qq*) and haplotype (*Q* and *q*) frequencies of each breed are shown in Table 2. The genotype and haplotype frequencies of the Japanese Black cattle population used in GWAS (named as Japanese Black cattle 1) were similar to those of the Japanese Black cattle population composed of unrelated animals (named as Japanese Black cattle 2). The different genotype and haplotype frequencies among these five breeds are also shown. For example, the frequencies of *QQ* genotype were 0.23 in Japanese Black cattle 2, 0.01 in Japanese Shorthorn cattle, 0.25 in Japanese Brown cattle, 0.09 in Angus cattle, and 0.04 in Holstein cattle.

**Table 2:** Genotype and haplotype frequencies of two non-synonymous SNPs in *ecto-5′-nucleotidase (NT5E)* in five different breeds

Breeds^a^	N	Genotype frequency			Haplotype frequency	
		*QQ*	*Qq*	*qq*	*Q*	*q*
Japanese Black cattle 1	574	0.17	0.49	0.34	0.42	0.58
Japanese Black cattle 2	542	0.23	0.46	0.31	0.46	0.54
Japanese Shorthorn cattle	109	0.01	0.21	0.78	0.11	0.89
Japanese Brown cattle	106	0.25	0.55	0.20	0.53	0.47
Angus cattle	118	0.09	0.44	0.47	0.31	0.69
Holstein cattle	204	0.04	0.28	0.67	0.19	0.81

^a^Japanese Black cattle 1, the population used in genome-wide association study; Japanese Black cattle 2, the population composed of unrelated animals and used for calculating genotype and haplotype frequencies

### Genetic architecture of IMP and its degradation products

In this population, the effects of the two non-synonymous SNPs in *NT5E* on IMP, inosine, and hypoxanthine were evaluated. The *Q* haplotype substitution effect and the proportions of genetic and phenotypic variances explained by the *Q* haplotype substitution effect are shown in Table 3. The genetic and phenotypic variances in each trait were estimated by single-trait animal model. The *Q* haplotype had negative effects on inosine (˗0.07) and hypoxanthine (˗0.12). The proportions of genetic and phenotypic variances for IMP, inosine, and hypoxanthine ranged from 0.30 to 0.46 and from 0.08 to 0.22, respectively. Moreover, the proportion of genetic variance for IMP was almost half of total genetic variance (0.46). In this study, we also evaluated the association of the two non-synonymous SNPs with other economically important traits, which were five carcass traits and 13 fatty acid compositions previously reported by Sasago et al. [16]. The results showed that no significant associations with *p*-value <0.01 were detected in these traits (Additional file 2: Table S2).

**Table 3:** Descriptive statistics, the results of genetic analysis, and the effect of *ecto-5′-nucleotidase (NT5E)* for inosine 5′-monophosphate (IMP) and its degradation products in beef

Traits^a^				Genetic analysis			*NT5E* effect		
	Descriptive statistics			Variance components^b^		Heritability	*Q* haplotype substitution effect	Proportion^c^	
	N	Mean	SD	Vg	Vp			Vg	Vp
IMP	571	0.40	0.21	0.018 (0.007)	0.038 (0.003)	0.48 (0.15)	0.13 (0.01)	0.46	0.22
Inosine	573	0.81	0.15	0.008 (0.003)	0.023 (0.002)	0.34 (0.14)	−0.07 (0.01)	0.30	0.10
Hypoxanthine	570	2.03	0.30	0.021 (0.011)	0.085 (0.006)	0.25 (0.13)	−0.12 (0.02)	0.33	0.08

^a^Unit is μmol/g meat. Standard errors are shown in parentheses

^b^V_g_, Genetic variance; V_p_, Phenotypic variance

^c^The proportion of V_g_ and V_p_ explained by the *Q* haplotype substitution effect

The genetic and phenotypic correlations among IMP, inosine, and hypoxanthine using the model (1) (see Materials and Methods) with and without *NT5E* effect as covariate are shown in Table 4. When the *NT5E* effect was included in the model (1), the genetic correlations of IMP with inosine and hypoxanthine increased (from ˗0.16 to 0.66 and from ˗0.72 to ˗0.49, respectively), and the genetic correlation of inosine with hypoxanthine decreased (from 0.53 to 0.19). The phenotypic correlations among the three traits did not show a large difference in the model (1) with and without *NT5E* effect, except for the phenotypic correlation of IMP with inosine (from 0.24 to 0.55).

**Table 4:** Genetic and phenotypic correlations estimated by the models with and without *ecto-5′-nucleotidase (NT5E)* effect

Trait	Model without *NT5E* effect^a^			Model with *NT5E* effect^a^		
	IMP	Inosine	Hx	IMP	Inosine	Hx
Inosine 5′-monophosphate (IMP)		−0.16	−0.72		0.67	−0.48
		(0.28)	(0.24)		(0.21)	(0.37)
Inosine	0.24		0.53	0.55		0.18
	(0.05)		(0.30)	(0.03)		(0.45)
Hypoxanthine (Hx)	−0.30	0.35		−0.17	0.27	
	(0.04)	(0.04)		(0.05)	(0.04)	

^a^Upper diagonal is genetic correlation and lower diagonal is phenotypic correlation. Standard errors are shown in parentheses

## Discussion

### GWAS, candidate gene analysis, and functional analysis

In this study, we investigated the genetic architecture of IMP and its degradation products in Japanese Black beef. First, we performed GWAS, candidate gene analysis, and functional analysis to detect the causal variants affecting IMP and its degradation products. The results of GWAS and candidate gene analysis showed that the three non-synonymous SNPs and the two SNPs in 3′-UTR in *NT5E* had high significance in these traits. In addition, no significant association of these traits was detected in loci outside of the *NT5E* locus. In functional analysis, the different enzymatic activity of NT5E was shown between *Q* and *q* allele of the two non-synonymous SNPs under in vitro conditions, when IMP is used as a substrate. In addition, the SNPs in 3′-UTR of *NT5E* did not affect the level of *NT5E* mRNA expression, which could not lead to an allelic imbalance. These results indicated that the two non-synonymous SNPs (c.1318C > T and c.1475 T > A) in *NT5E* affect the amount of IMP, inosine, and hypoxanthine in beef by regulating enzymatic activity.

The detailed studies on the quantitative trait loci (QTL) affecting IMP and its degradation products have not been reported in livestock population. On the other hand, significant associations between *NT5E* and some traits have been reported recently in humans. For example, the serum inosine concentration is the biomarker of metabolic traits involved in purine metabolic pathways [12]. Recently, the metabolome-wide GWAS was performed to evaluate the genetic variance in comprehensive human metabolisms [22]. The result showed that a significant association exists between an SNP near *NT5E* and inosine concentration in human serum. Another example is the arterial and joint calcifications, an extremely rare mendelian disorder associated with increased cardiovascular risk, the genetic architecture of which was unclear [23]. Hilaire et al. [23] performed linkage analysis and showed that rare mutations in *NT5E* affect the arterial and joint calcifications due to loss of NT5E function. Zhang et al. [24] also performed candidate gene analysis and showed the association between calcification of joints and arteries and another non-synonymous SNP in *NT5E* (p.Gly454Arg), which was far away from the binding site of substrate AMP but next to the lacked locus of shorter NT5E isoform known as CD73S [25].These results indicated that the mutations in *NT5E* would affect the enzymatic activity of NT5E and thus were associated with these traits. The objective of finding variants associated with these traits are different in between human (for health) and cattle (for breeding), but our results could contribute to the genetic understanding the effect of *NT5E* variants on both human and cattle nucleotide metabolisms.

### Bioinformatics analysis of two-synonymous SNPs

The two non-synonymous SNPs (c.1318C > T and c.1475 T > A) were under complete LD in five different breeds. The result indicated the difficulty in evaluating the effect of SNP by separating the two SNPs and obtaining more than two types of haplotypes in vivo. The c.1475 T > A SNP encodes a highly conserved amino acid in mammals, and the predicted 3D protein structure showed that the SNP (p.Val492Glu) is located on the β-sheet for binding IMP ligand. Therefore, c.1475 T > A SNP could have a direct effect on enzyme–substrate binding. As for c.1318C > T, it encodes amino acid which is not conserved in some ruminants (goat and sheep), but the region flanking the amino acid is highly conserved in mammals. In addition, the SNP (p.His440Tyr) is far away from the binding site of the IMP ligand. Thus, c.1318C > T may not have a direct effect on enzyme–substrate binding. However, it could affect the enzymatic activity of NT5E from the non-binding site. For example, human *NT5E* has two splice variants, which encode full-length canonical NT5E protein and shorter NT5E isoform known as CD73S [25]. Human CD73S lacks 5′-nucleotidase activity, because this enzyme lacks amino acids 404–453 in exon 7 located in the C-terminus. The C-terminus contains the important interface for forming the functional NT5E homodimer expressed on the cell surface [20]. The c.1318C > T is located on the absent locus of exon 7 (corresponding to 440th amino acid in human NT5E). Therefore, c.1318C > T could also affect the formation of the functional NT5E homodimer, and thus affect the enzymatic activity of NT5E.

### Genetic architecture of IMP and its degradation products

**Fig. 6:**
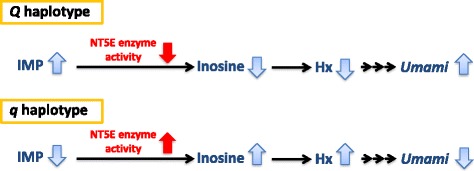
Proposed model for the difference of ecto-5′-nucleotidase (NT5E) enzyme activity between *Q* and *q* haplotypes in beef. IMP, inosine 5′-monophosphate; Hx, hypoxanthine

In this study, two QTNs (c.1318C > T and c.1475 T > A) in *NT5E* contributed to about half of the total genetic variance in IMP. The *Q* haplotype with positive effect on IMP has a negative effect on its enzymatic activity in IMP degradation, and thus contributes to increase *umami* taste (Fig. 6). In addition, the genetic correlation of IMP with inosine largely increased when *NT5E* effect was included as a covariate in the model (1). These results indicated that the QTNs in *NT5E* strongly affected the enzymatic activity of NT5E and thus strongly affects the overall balance in concentration of IMP and its degradation products in beef.

The changes in the amount of IMP and its degradation products in beef are influenced by postmortem conditioning and the aging period [14, 15]. The overall change in concentration of IMP and its degradation products strongly depend on the aging period until 20 days after slaughter [14, 15]. After 20 days, the amount of IMP remains almost unchanged [15]. Some of the enzymes involved in IMP degradation are gradually inactivated after 20 days, since the proteolytic activity in postmortem muscle affects other enzymatic processes, such as glycolysis [26]. The IMP in muscle is degraded by 5′-nucleotidase in intracellular and NT5E in extracellular (Fig. 1). The aging period after slaughter of cattle induces structural changes in the cell membrane and leads to intracellular water efflux [13]. The intracellular fluid could lead to an increase the amount of IMP in extracellular during aging, and the NT5E then involves the degradation of IMP in extracellular. In this study, no significant association of IMP and its degradation products was detected in loci outside of the *NT5E* locus by GWAS. The result indicated that the enzymatic activity of NT5E strongly affects the difference in IMP degradation in beef.

Some of the major QTLs affecting the economically important traits are still segregated in a population, even if cattle populations go through intensive artificial selection for the traits. However, such QTLs with a favorable effect on the traits may have an unfavorable effect on other economical traits. For example, the allele in the *Acyl-CoA:diacylglycerol acyltransferase 1* (*DGAT1)* increases milk fat content and decreases milk yield in dairy cattle [27], and the risk allele *FYVE, RhoGEF and PH domain-containing protein 3 (FGD3)* of skeletal dysplasia increases carcass weight [28]. Therefore, it is difficult to apply marker-assisted selection (MAS) using these QTLs. On the other hand, unselected traits, which have the potential for future breeding objectives, such as meat quality traits, could retain high genetic variance. A major QTL affecting the traits might not have an unfavorable effect on other selection traits. IMP is currently not under selection in Japanese Black cattle breeding, but the *NT5E* is a major QTL affecting the IMP in beef and is not associated with carcass traits and fatty acid compositions. In addition, the detected QTNs in *NT5E* were common variants and different haplotype frequency was seen among different breeds. The frequency of favorable *Q* haplotype was greater in Japanese Black cattle than in other breeds such as Angus cattle and Holstein cattle. Therefore, IMP could be a future objective trait in Japanese Black cattle, and *NT5E* could be useful as a means of improving meat quality by MAS.

## Conclusion

The overall change in concentration of IMP and its degradation products in meat can potentially affect the beef palatability and especially *umami* taste. We investigated the genetic architecture of these traits to utilize them in Japanese Black cattle breeding to enhance meat quality. Our study demonstrated that two non-synonymous SNPs in *NT5E* affect the amount of IMP and its degradation products in meat by regulating NT5E enzymatic activity. The genetic architecture of IMP and its degradation products included the *NT5E* with a very large effect, and the QTNs in *NT5E* affected the overall concentration balance of IMP and its degradation products. In addition, the QTNs in *NT5E* were common variants in different breed populations and did not have an unfavorable effect on the carcass traits and fatty acid composition. Based on all the above findings taken together, IMP could be a breeding target in the future for increasing *umami* taste in beef, and the QTNs in *NT5E* could be useful for improving meat quality by MAS in Japanese Black cattle.

## Methods

### Animals, phenotypes, and genotyping for GWAS

The complete description of the population was reported in the previous study [8, 16]. A commercial Japanese Black cattle population, produced in Yamagata Prefecture, Japan, was used in this study. *Longissimus thoracis* muscles were collected from 574 Japanese Black cattle slaughtered at a meat processing plant in Yamagata Prefecture from 2011 to 2013. The muscles located at the 7th thoracic vertebra (frozen 16–19 d after slaughter) were purchased from a distributor. The muscles were thawed at 2 °C for 16 h and were then cut for nucleotide measurements. The samples were then stored at ˗30 °C until analysis.

IMP, inosine, and hypoxanthine were measured in this study. Phenotypes that were not within the mean ± 3 standard deviation (SD) range for each trait were considered outliers and were deleted. The descriptive statistics of these traits are shown in Table 3. The details on the procedure for measuring the traits in this study have been described by Sakuma et al. [8]. The extractions of nucleotides were performed using approximately 0.10 g samples of meat taken from the minced raw meat (about 15 g in total). The sample was homogenized with ultrapure water and N-hexane, with cytidine solution as an internal standard. The underlayer was obtained after the removal of fat and protein using hexane and acetonitrile, respectively. The supernatant was filtered through a 0.45-μm microfilter (Millex-LH, Merck Millipore, Billerica, USA), and the filtrate was then mixed with 45% acetonitrile solution. Approximately 20 μL of the filtrate was mixed with 180 μL of ultrapure water, and the resulting solution was analyzed using the high performance liquid chromatography (Waters 2695, Waters, Milford, USA), along with an Atlantis T3 column (4.6 × 150 mm, 5 μm, Waters) and a UV detector (Waters 2487, Waters). The nucleotides were identified by comparing their retention times with those of the established standards. The concentrations of each were calculated using internal and external standard solutions, expressed as μmol per gram of meat.

The complete description of the SNP array genotyping was reported in a previous study by Sasago et al. [16]. The genomic DNA of 574 animals was briefly extracted from muscle samples using phenol-chloroform extraction. The DNA samples were genotyped using the Illumina BovineSNP50 v2 BeadChip (Illumina, CA, USA) and the GenomeStudio software (Illumina, CA, USA). The SNP maps were updated according to the SNPchiMpv.3 database [29] and the UMD3.1 assembly, and autosomal chromosomes were used in this study. The SNP quality control was assessed using PLINK software [30]. The exclusion criteria for SNPs included a minor allele frequency (MAF) < 0.01, a call rate < 0.95, and a Hardy-Weinberg equilibrium test with *p*-value <0.001. A total of 40,657 SNPs on the array were used in the present study.

### Genetic analysis

The genetic parameters of IMP, inosine, and hypoxanthine in meat were estimated by the following animal model

1 $$ {\displaystyle \begin{array}{l}{y}_{ik jlmno}={\mu}_i+{sex}_{ij}+{year}_{ik}+{month}_{il}+{aging}_{im}+{farm}_{in}\\ {}\kern4em +{b}_1{x}_{ij klmno}+{b}_2{x}_{ij klmno}^2+{u}_{ij klmno}+{e}_{ij klmno}\end{array}} $$

where *y*_*ijklmno*_ is the observation of the animal *o* for trait *i*; μ_i_ is the total mean for trait *i*; *sex*_*ij*_ is the fixed effect of sex *j* (2 classes) for trait *i*; *year*_*ik*_ is the fixed effect of the slaughter year *k* (3 classes, 2011–2013) for trait *i*; *month*_*il*_ is the fixed effect of the slaughter month *l* (12 classes) for trait *i*; *aging*_*im*_ is the fixed effect of aging period *m* (4 classes, 16–19 days) for trait *i*; *farm*_*in*_ is the fixed effect of the farm *n* (13 classes) for trait *i*; ${b}_1{x}_{ijklmno}+{b}_2{x}_{ijklmno}^2$ are the linear (*b*_*1*_) and quadratic (*b*_*2*_) regression coefficients on slaughter age (*x*_*ijklmno*_) for trait *i*; *u*_*ijklmno*_ is the random additive genetic effect of animal *o* for trait *i*; *e*_ijklmno_ is the random residual effect for trait *i*. The pedigrees were traced back to five generations, and a total of 3513 animals were used in this study. Single-trait animal model for estimating heritability and multi-trait animal model for estimating genetic and phenotypic correlations were applied using model (1). The ASReml 3.0 software [31] was used to estimate (co)variance components with standard errors and best linear unbiased estimators (BLUEs) of all fixed effects. The results of estimated variance components and heritability for three traits by single-trait animal model are shown in Table 3.

### GWAS

Firstly, the phenotypic values were adjusted using any fixed non-genetic effects in model (1). The BLUEs for all fixed effects were obtained by single-trait animal model, and the adjusted phenotype was calculated by subtracting the BLUEs from raw phenotypic values. Secondly, the vectors of adjusted phenotypes (**y**_**adj**_) were used as dependent traits in a linear mixed model approach for each SNP as follows:

2 $$ {\mathbf{y}}_{\mathbf{adj}}={\beta}_i{\mathbf{w}}_i+\mathbf{a}+\mathbf{e}{\hbox{'}}_i $$

where *β*_*i*_ is the allele substitution effect of the effect allele, **w**_*i*_ is a vector of the SNP genotypes (coded as 0, 1, or 2 for the homozygote, heterozygote, and the other homozygote, respectively), and **e′**_*i*_ is a vector of the random residual effect at the *i*-th SNP distributed as $N\left(0,\mathbf{I}{\sigma}_{e\hbox{'}}^2\right)$, where **I** and ${\sigma}_{e\hbox{'}}^2$ are the identity matrix and residual variance, respectively. **a** is a vector of random genetic effects ($\mathbf{a}\sim N\left(0,\mathbf{G}{\sigma}_a^2\right)$), where **G** and ${\sigma}_a^2$ are the genomic relationship matrix proposed by VanRaden [32] and the SNP genetic variance, respectively. The regression coefficient and *p*-values tested by Wald test were obtained using GEMMA software [33]. The proportion of phenotypic variance explained by the *i*-th SNP effect was calculated using the formula

$$ {Proportion}_i=\frac{2{p}_i\left(1-{p}_i\right){\beta}_i^2}{V} $$

where *p*_*i*_ is MAF of the *i*-th SNP [34], and *V* is the genotypic (V_g_) or phenotypic (V_p_) variances obtained in a single-trait animal model in genetic analysis (Table 3). The Bonferroni correction was applied to determine the 5% genome-wide significance thresholds (*p* = 1.37 × 10^−6^). The genes within ±50 kb of the significant SNPs were scanned using the NCBI2R R-package. The values of r^2^ between individual SNPs were calculated, and the haplotype block pattern was visualized using Haploview 4.0 software [35].

### NT5E sequencing and its effect

We used the genomic DNA to determine the nucleotide sequences of *NT5E* to detect its variants. Each of the eight animals was selected from the most extreme upper and lower residuals of IMP in the population. The residuals in this analysis were calculated by subtracting the additive genetic effect and all the non-genetic effects in model (1) from phenotypic values. For the analysis of polymorphisms in *NT5E*, the fragments of the full-length exon region and proximal promoter region (upstream region from the start codon to 1591 bp upper region) were directly sequenced with 15 primer sets (Additional file 3: Table S3). The primer sets were designed by PRIMER3 software [36] according to the information about bovine *NT5E* mRNA and Btau4.6 genome sequences (GenBank accession no. NM_174129.3 and NC_007307, respectively), because the *NT5E* was not correctly assigned in UMD3.1 assembly (GeneBank accession no. AC_000166). Polymerase chain reaction (PCR) was performed in a 15-μL volume of solution containing 20 ng of genomic DNA, 6.25 pmol of each primer, 0.2 mM of each deoxynucleoside triphosphate, 10 mM Tris-HCl (pH 8.3), 50 mM KCl, 1.5 mM MgCl_2_, and 0.375 U of KOD-FX DNA polymerase (Toyobo, Osaka, Japan). The PCR was performed as follows: 94°C for 2 min followed by 30 cycles of 98°C for 10 s, 55°C for 30 s, and 68°C for 40 s. The PCR products were purified using the ExoSAP-IT PCR Product Cleanup (USB Corporation, Cleveland, OH, USA) and then directly sequenced using the ABI PRISM 3130 DNA Sequencer and Sequencing Analysis 3.4 software (Applied Biosystems Japan, Tokyo, Japan). The polymorphisms were then checked, and the reference SNP ID number was obtained through the dbSNP database (http://www.ncbi.nlm.nih.gov/SNP). A total of eight SNPs and one indel variant, including one synonymous SNP, were identified in this population, and all variants were present in dbSNP database. The eight variants except for one synonymous SNP were then genotyped in all animals by direct sequencing as shown above.

The association tests of all variants in *NT5E* with three traits were performed using the model (2). In addition, estimation of genetic and phenotypic correlations was performed using the model (1), which included the c.1475 T > A SNP as a covariate in the model (1). As for the most significant SNP, the association test of c.1475 T > A SNP with other economically important traits was also performed using the model (2). The economically important traits used in this study were five carcass traits and 13 fatty acid compositions described by Sasago et al. [16] (Additional file 2: Table S2).

### Expression construct for NT5E

To measure the enzymatic activity of NT5E, the coding region of *NT5E* (NM_174129.3) of each haplotype (*Q* and *q* haplotype) was PCR amplified using PrimeSTAR Max DNA Polymerase (Takara, Cat. #R045A) from cDNA derived from the dermal primary fibroblasts using a forward primer (5′- atgaatcccggagcggctcgcaccccggcgctgaggatcctcgcgctgggcgcgttgctgtggcccgcggcgcgccccATGGGATCCTACCCTTACGACGTTCCTGATTACGCTAGCCTCGAATTCtgggagctcaccatcttgcacacc-3′; lowercase letters + underline indicate the signal sequence, and uppercase letters indicate hemagglutinin [HA] tag), and a reverse primer (5′- ATAAGAATGCGGCCGCctattggtataaaataatgatc-3′; uppercase letters indicate the *Not*I linker). The PCR product was cloned into the blunted *EcoR*I and *Not*I sites of the pCAGGS vector [37]. The sequence and orientation of the insert were confirmed by sequencing. The expression of NT5E was confirmed by western blotting with an anti-HA antibody 3F10 (Roche, Cat. #11867423001, 100 ng/mL). Immunoreactivity was detected with a horseradish peroxidase-conjugated donkey anti-rat IgG antibody (Jackson ImmunoResearch, Cat. #712–035-153) and the ECL Prime Western Blotting Detection Reagent (GE Healthcare, Cat. #RPN2232). Chemiluminescence was detected with an ImageQuant LAS 4000 (GE Healthcare) and quantified using the ImageQuant TL Analysis Toolbox.

To determine whether non-synonymous SNPs affect the enzymatic activity of NT5E, pCAGGS-*NT5E* (*q* haplotype) was subjected to site-direct mutation using the following primers: c.1318C > T (forward primer: 5′- gagcacagcgtgTaccgctatggccaggccac-3′, reverse primer: 5′- tagcggtAcacgctgtgctcgaaggccttcttc-3′); c.1475 T > A (forward primer: 5′- gaatggataaggAgtacaaggtgatcctccc-3′, reverse primer: 5′- cttgtacTccttatccattctaagaggctc-3′); and c.1526A > G (forward primer: 5′- gagacggattccGgatgataaaagatgaaaag-3′, reverse primer: 5′- tatcatcCggaatccgtctccaccactgacaag-3′). Uppercase letters indicate the mutation allele (*Q* allele). After the PCR amplification with PrimeSTAR Max DNA Polymerase, the PCR products were digested with *Dpn*I (Toyobo, Cat. #DPN-101) and were then transformed into One Shot TOP10 Competent *E. coli* (ThermoFisher, Cat. #C404003). The sequence were confirmed by sequencing.

### IMPase activity assay

To determine whether the haplotype and non-synonymous SNPs in the exon 7 and exon 8 affect the enzymatic activity of NT5E in COS-7 cells, we transfected 0.4 × 10^5^ cells per well into a 24-well plate with a mixture of 0.4 ng of the pCAGGS-NT5E. COS-7 cells were used in this study as it exhibits negligible endogenous nucleotide-metabolizing enzyme activities [38, 39]. About 48 h after the transfection, the cells were washed twice with saline. The IMP activity of the intact cells was determined by measuring the Pi liberated as a result of the degradation of 1 mM IMP (Sigma, Cat. # I4625) for 30 min using Malachite Green Phosphate Assay Kit (echelon, Cat. #K-1500) and plate reader (Bio-Rad, Cat. #iMark). The results were tested by analysis of variance (one-way ANOVA) followed by the Tukey HSD (honestly significant difference) multiple comparisons test for significant difference among the five constructs (Q-Q-Q, q-q-q, Q-q-q, q-Q-q, and q-q-Q).

### Allelic imbalance test

To quantify the potential allelic imbalance of *NT5E* transcripts, we designed PCR primers to c.1475 T > A in exon 8 of *NT5E*. The forward primer was 5′- gggacagggtggtcaagtta −3′, and the reverse primer was 5′- cagagtcatgttttatcttttcatctt −3′. A total of 14 skeletal muscles from heterozygotes in Japanese Black cattle in NLBC were collected. We used 50 ng of template cDNA from skeletal muscles or 10 ng of genomic DNA from heterozygous animals for PCR amplification with TaKaRa Ex Taq HS DNA Polymerase (TaKaRa, Cat. #RR006). The PCR product was directly sequenced and purified using the CleanSEQ system (Agencourt, Cat. #A29154). The peak heights at the polymorphic sites were quantified using PeakPicker 2 software [17]. Allelic imbalances were estimated as the ratio of the peak height of the *Q* allele to that of the *q* allele in cDNA and in genomic DNA from the same animal. Calibration curves were generated using data obtained by mixing varying amounts of genomic DNA from *Q* and *q* homozygotes. Welch’s *t*-test was performed to compare the differences between the ratios of the peak height of the *Q* allele over the *q* allele in genomic DNA and cDNA.

### Bioinformatics analysis

For the three non-synonymous SNPs detected in the exon 7 and exon 8, multiple sequence alignments for *NT5E* were conducted using Clustal W [40] using cow, mouse, rat, human, goat, sheep, and horse genomic sequences. To illustrate the effects of the three non-synonymous SNPs, the 3D structure of NT5E reference sequence (NP_776554.2) was modeled by the homology modeling using the SWISS-MODEL server [18, 19]. The template structure of human NT5E, which is known as CD73, crystal form I (4h2f), crystal form II (4h2g), and crystal form III (4h2i) [20] from protein data bank (PDB) [41] were applied for the modeling. We used GMQE and QMEAN4 scores to discriminate the good model from all other models (higher numbers indicate higher reliability). The template structure of NT5E crystal form II (4h2g) had the highest values of GMQE (0.95) and QMEAN4 (0.53) in the SWISS-MODEL server (Additional file 4: Table S4). The structural model based on the template structure of NT5E crystal form II was then visualized by Swiss-PdbViewer software [42]. The docking of the obtained structural model with the 3D structure of IMP ligand (ZINC database ID: 14,951,284) [43] was carried out with SWISSDOCK server based on EADock DSS [21], because the NT5E crystals in PDB were not obtained in the presence of IMP ligand. A total of 42 binding clusters were generated in the vicinity of all target cavities, and the top five most favorable clusters (Additional file 5: Table S5) were then visualized by the Swiss-PdbViewer software.

### Genotype and haplotype frequencies of two non-synonymous SNPs in five different breeds

We obtained blood samples from Japanese Black cattle (*n* = 542), Japanese Shorthorn cattle (*n* = 109), Japanese Brown cattle (*n* = 106), Angus cattle (*n* = 118), and Holstein cattle (*n* = 204) raised at Tokachi, Ohu, Iwate, Tottori, Miyazaki, and Kumamoto stations of NLBC, respectively, in order to screen for the two non-synonymous SNPs. Japanese Black cattle, Japanese Shorthorn cattle, and Japanese Brown cattle are beef cattle called “Wagyu” [44], with different genetic background [45]. For Japanese Black cattle and Holstein cattle, the samples were selected using the criteria of at most 5 progenies in each sire, and these animals were low relatives with the progeny of 164 sires and 110 sires, respectively. The genomic DNA was extracted using phenol-chloroform extraction. The two non-synonymous SNPs were genotyped using the Cycleave PCR system (Takara Bio Inc., Shiga, Japan), and the PCR reaction mixture was prepared using Premix Ex Taq™ (Probe qPCR; TaKaRa Bio Inc.). Genotyping was done using an ABI StepOnePlus Real-Time PCR system (Applied Biosystems), and the primer and the probe information are shown in Additional file 6: Table S6. The reaction conditions were 95 °C for 20 s; 40 cycles at 95 °C for 1 s and 60 °C for 20 s; and 60 °C for 10 s.

## Additional files

Additional file 1: Table S1. Significant genome-wide single nucleotide polymorphisms (SNPs) for inosine 5′-monophosphate (IMP), inosine, and hypoxanthine in meat. (XLSX 16 kb)

Additional file 2: Table S2. Association test of *ecto-5′-nucleotidase* (*NT5E*) with carcass traits and fatty acid compositions. (XLSX 12 kb)

Additional file 3: Table S3. Sequences of PCR primers used to amplify and sequence the genomic DNA. (XLSX 10 kb)

Additional file 4: Table S4. Details of the parameters for three selected models of human ecto-5′-nucleotidase in SWISS-MODEL server. (XLSX 9 kb)

Additional file 5: Table S5. Top five clustering results obtained from the docking of inosine 5′-monophosphate into ecto-5′-nucleotidase by SWISSDOCK server. (XLSX 9 kb)

Additional file 6: Table S6. Primer and reporter information for the two non-synonymous SNP genotyping. (XLSX 15 kb)

## Abbreviations

ADP: Adenosine diphosphate
AMP: Adenosine monophosphate
ATP: Adenosine triphosphate
BLUE: Best linear unbiased estimator
DGAT1: Acyl-CoA:diacylglycerol acyltransferase 1
FGD3: FYVE, RhoGEF and PH domain-containing protein 3
GWAS: Genome-wide association study
IMP: Inosine 5′-monophosphate
LD: Linkage disequilibrium
MAS: Marker-assisted selection
NT5E: Ecto-5′-nucleotidase
PCR: Polymerase chain reaction
PDB: Protein data bank
QTL: Quantitative trait locus
QTN: Quantitative trait nucleotide
SD: Standard deviation
SNP: Single nucleotide polymorphism
UTR: Untransrated region
